# Impact of national holidays and weekends on incidence of acute type A aortic dissection repair

**DOI:** 10.1038/s41598-022-25076-7

**Published:** 2022-11-29

**Authors:** Anna Oudin, Henrik Bjursten, Daniel Oudin Åström, Shahab Nozohoor, Khalil Ahmad, Mariann Tang, Markus Bjurbom, Emma C. Hansson, Anders Jeppsson, Christian H. Moeller, Mikko Jormalainen, Tatu Juvonen, Ari Mennander, Peter S. Olsen, Christian Olsson, Anders Ahlsson, Emily Pan, Peter Raivio, Anders Wickbom, Johan Sjögren, Arnar Geirsson, Tomas Gudbjartsson, Igor Zindovic

**Affiliations:** 1 Division of Occupational and Environmental Medicine, Department of Laboratory Medicine, Lund University, Lund, Sweden; 2 Division of Sustainable Health, Department of Public Health and Clinical Medicine, Umeå University, Umeå, Sweden; 3 Department of Cardiothoracic Surgery, Skåne University Hospital, Lund University, Lund, Sweden; 4 Department of Cardiothoracic and Vascular Surgery, Aarhus University Hospital, Skejby, Denmark; 5 Department of Thoracic and Cardiovascular Surgery, Karolinska University Hospital, Stockholm, Sweden; 6 Department of Molecular and Clinical Medicine, Institute of Medicine, Sahlgrenska Academy, University of Gothenburg, Gothenburg, Sweden; 7 Department of Cardiothoracic Surgery, Sahlgrenska University Hospital, Gothenburg, Sweden; 8 Department of Cardiothoracic Surgery, The Heart Centre, Rigshospitalet, Copenhagen University Hospital, Copenhagen, Denmark; 9 Heart and Lung Center, Helsinki University Hospital, Helsinki, Finland; 10 Heart Centre, Tampere University Hospital and University of Tampere, Tampere, Finland; 11 Heart Center, Turku University Hospital, Turku, Finland; 12 Brigham and Women’s Hopistal and Harvard Medical School, Boston, MA USA; 13 Department of Cardiothoracic and Vascular Surgery, Örebro University Hospital, Örebro, Sweden; 14 Division of Cardiac Surgery, Yale University School of Medicine, New Haven, CT USA; 15 Department of Cardiothoracic Surgery, Landspitali University Hospital and Faculty of Medicine, University of Iceland, Reykjavik, Iceland

**Keywords:** Medical research, Epidemiology

## Abstract

Previous studies have demonstrated that environmental and temporal factors may affect the incidence of acute type A aortic dissection (ATAAD). Here, we aimed to investigate the hypothesis that national holidays and weekends influence the incidence of surgery for ATAAD. For the period 1st of January 2005 until 31st of December 2019, we investigated a hypothesised effect of (country-specific) national holidays and weekends on the frequency of 2995 surgical repairs for ATAAD at 10 Nordic cities included in the Nordic Consortium for Acute Type A Aortic Dissection (NORCAAD) collaboration. Compared to other days, the number of ATAAD repairs were 29% (RR 0.71; 95% CI 0.54–0.94) lower on national holidays and 26% (RR 0.74; 95% CI 0.68–0.82) lower on weekends. As day of week patterns of symptom duration were assessed and the primary analyses were adjusted for period of year, our findings suggest that the reduced surgical incidence on national holidays and weekends does not seem to correspond to seasonal effects or surgery being delayed and performed on regular working days.

## Introduction

National holidays and different days of the week have previously been hypothesised to affect both cardiovascular mortality and occurence of acute aortic dissection (AAD)^1–5^. For example, in a Swedish study of 283,014 cases of myocardial infarction from 1998 to 2013, a higher risk of myocardial infarction was observed during Christmas, New Year and Midsummer holidays, but not during the Easter holiday ^1^. However, in an Australian study investigating 700,000 stroke deaths and 250,000 cardiovascular deaths, there was no increased risk of cardiovascular or stroke mortality during Christmas. In these studies, the authors observed that the winter months showed a higher mortality and speculated that international differences in holiday effects may relate to temperature, culture or medical systems^2^.

In a study of 957 patients who had suffered an AAD, the frequency of AAD was significantly higher during winter but only among patients aged < 70 years, those with type B AAD and those without hypertension or diabetes^3^. Furthermore, in a Japanese study of 435 patients, it was shown that AAD occurred more frequently in the morning hours and during winter^4^; and in a Chinese study of 1121 patients from the Hebei Province, it was shown that AAD was more common in winter than in summer and that there was a clear circadian pattern, where onset of the disease peaked between 13:00 and 18:00^5^. An incidence of ATAAD of approximately 2 -16 cases per 100,000 persons has been reported^6^, and in a recent German study, emergency department incidences varied between 5.9/100,000 and 24.9/100,000^7^. However, the incidence patterns of acute type A aortic dissection (ATAAD) repair in relation to national holidays and weekends have not been investigated. 

Therefore, the aim of the present study was to investigate whether the incidence of ATAAD repair is influenced by national holidays and weekends in a large, relatively homogenic Nordic contemporary cohort of patients undergoing ATAAD repair.

## Material and methods

### Data

For the time period 1st of January 2005 to 31st of December 2019, we collected daily data on counts of ATAAD repairs for each of the 10 participating university hospitals, eight of which were included in the original NORCAAD collaboration (Reykjavik, Iceland; Aarhus and Copenhagen in Denmark; Gothenburg, Lund, Stockholm and Örebro in Sweden; and Tampere and Turku in Finland) with the addition of the university hospitals in Copenhagen and Helsinki. In total, we included 2995 ATAAD repairs performed during the study period. The NORCAAD registry has been described in detail elsewhere^8^. The study was approved by the national or regional review boards of each participating center (*i.e.* The Swedish ethical review authority; The regional research committee, Region Hovedstaden; The regional research committee, Region Nordsjaelland; Tampere University Hospital Ethics Committee; The Ethics Committee of the Hospital District of Southwest Finland; The research ethics committee of the Faculty of Medicine, University of Helsinki and The University of Iceland Science Ethics Committee. Informed patient consent was waived by above mentioned each of the review boards.

### Definitions

An ATAAD was defined as an aortic dissection with symptom duration less than 14 days. Weekends occurred during Saturdays and Sundays and national holidays were defined as nation-specific, centrally established holidays on which most people do not have to work and healthcare institutions do not maintain their regular activities. Saturdays and Sundays occurring during national holidays were included in both analyses.

### Statistical methods

#### National holiday/weekend—ATAAD association

The association between national holidays, weekends and ATAAD was investigated by first fitting Poisson regression models for each country, as national holidays may not occur at the same dates throughout the investigated countries. National holidays were thus country-specific. We adjusted the models for dispersion and month of the year and included an interaction term between national holidays and weekend. In the second step, we fitted a fixed-effect meta-regression to pool the country-specific estimates into a region-specific estimate. To investigate whether season modifies the effect of national holidays and weekends on outcome, we stratified the analyses to investigate the three coldest months (December, January and February) separately. Post hoc analyses of symptom duration for weekdays was based on the Kruskal–Wallis test and Mann–Whitney U-test for analysing national holidays. Additional post hoc analyses were performed using the Chi-squared test to ascertain no systematic differences in the distribution of operations between patients with available information about symptom duration compared to those where this data was not available. We calculated the E-value for the meta-analytic measures to investigate the strength an unmeasured confounder would have in order to be associated with both the outcome as well as the exposure to discard the observed association^9^. R version 4.0.3^10^ and package meta^11^ was used in the analyses and a *p* value < 0.05 was regarded as statistically significant.

### Ethical approval

The study was approved by the regional or institutional review board according to national guidelines for approval of registry studies and the methods were carried out in accordance with relevant guidelines and regulations. No experiments were conducted and thus, no experimental protocols were reviewed.

## Results

A total of 2995 operations for ATAAD were performed at the participating centres during the study period. Table 1 shows the annual counts of ATAAD operations for the included centres.

**Table 1:** Annual numbers of ATAAD repairs for the participating centres.

Centre	Annual number of ATAAD repairs
	Median (IQR)
**Denmark**	
Aarhus	15 (10–22)
Copenhagen	44 (31–51)
**Finland**	
Helsinki	22 (19–25)
Tampere	9 (5–11)
Turku	7 (5–10)
**Iceland**	
Reykjavik	3 (2–4)
**Sweden**	
Gothenburg	33 (30–35)
Lund	24 (22–28)
Stockholm	29 (25–32)
Örebro	11 (9–14)

ATAAD, Acute Type A Aortic Dissection; IQR, Interquartile Range.

**Figure 1:**
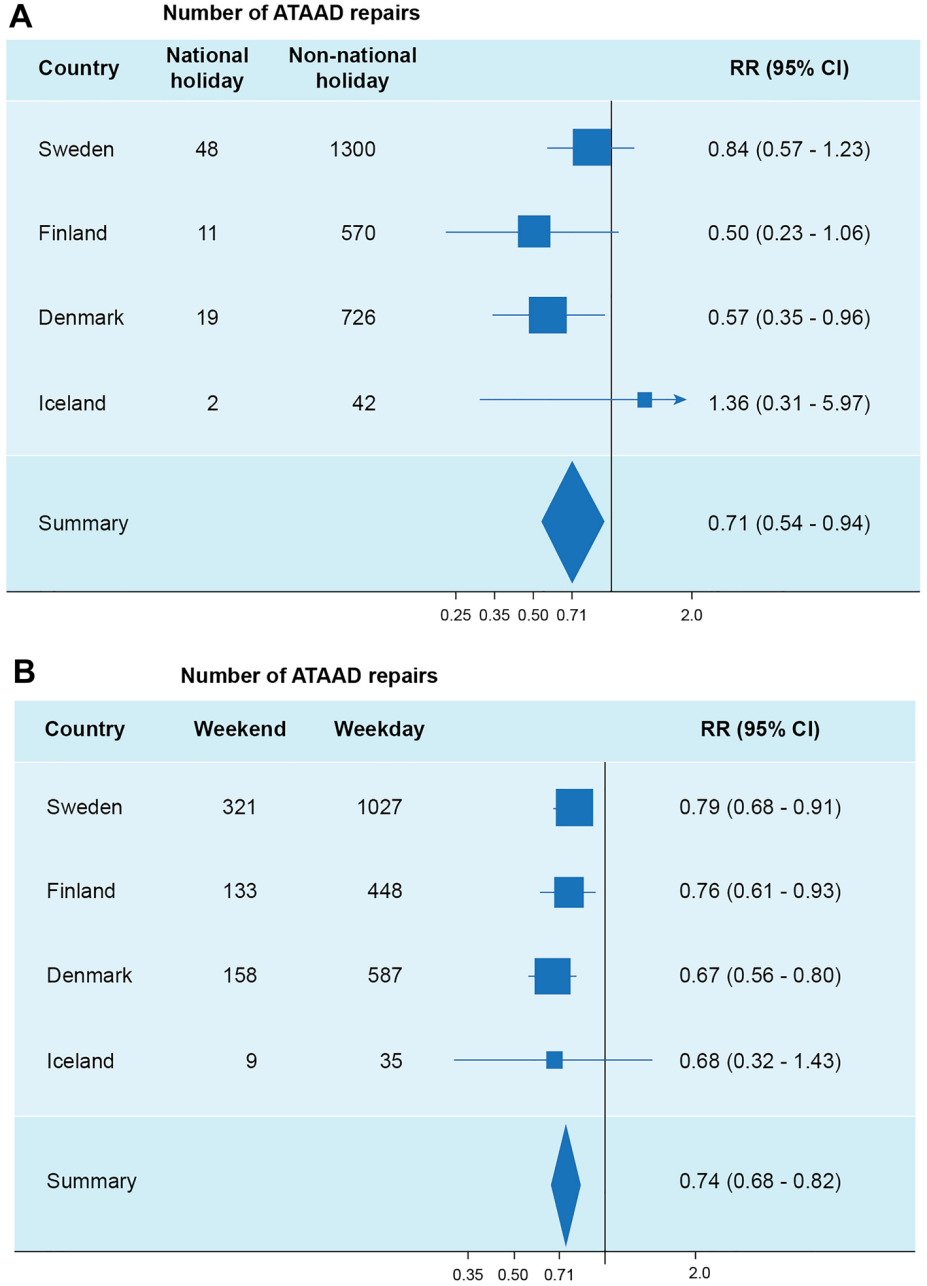
Forest plot illustrating the relative risk of surgery for ATAAD on national holidays (NH) (**a**) and weekends (w-end) and weekdays (w-days) (**b**) presented per country and pooled.

Figure 1a,b illustrate the country-specific and pooled relative risks (RR) and 95% CIs for the association between national holidays, weekends and ATAAD repair. We observed significantly lower relative risks of ATAAD repairs on both nation-specific national holidays and weekends for the pooled estimates. Compared to other days, the number of ATAAD repairs was lower on national holidays and weekends: 29% (RR 0.71; 95% CI 0.54–0.94) and 26% (RR 0.74; 95% CI 0.68–0.82), respectively. The meta-estimates were similar between the cold season and the rest of the year (results not shown).

**Figure 2:**
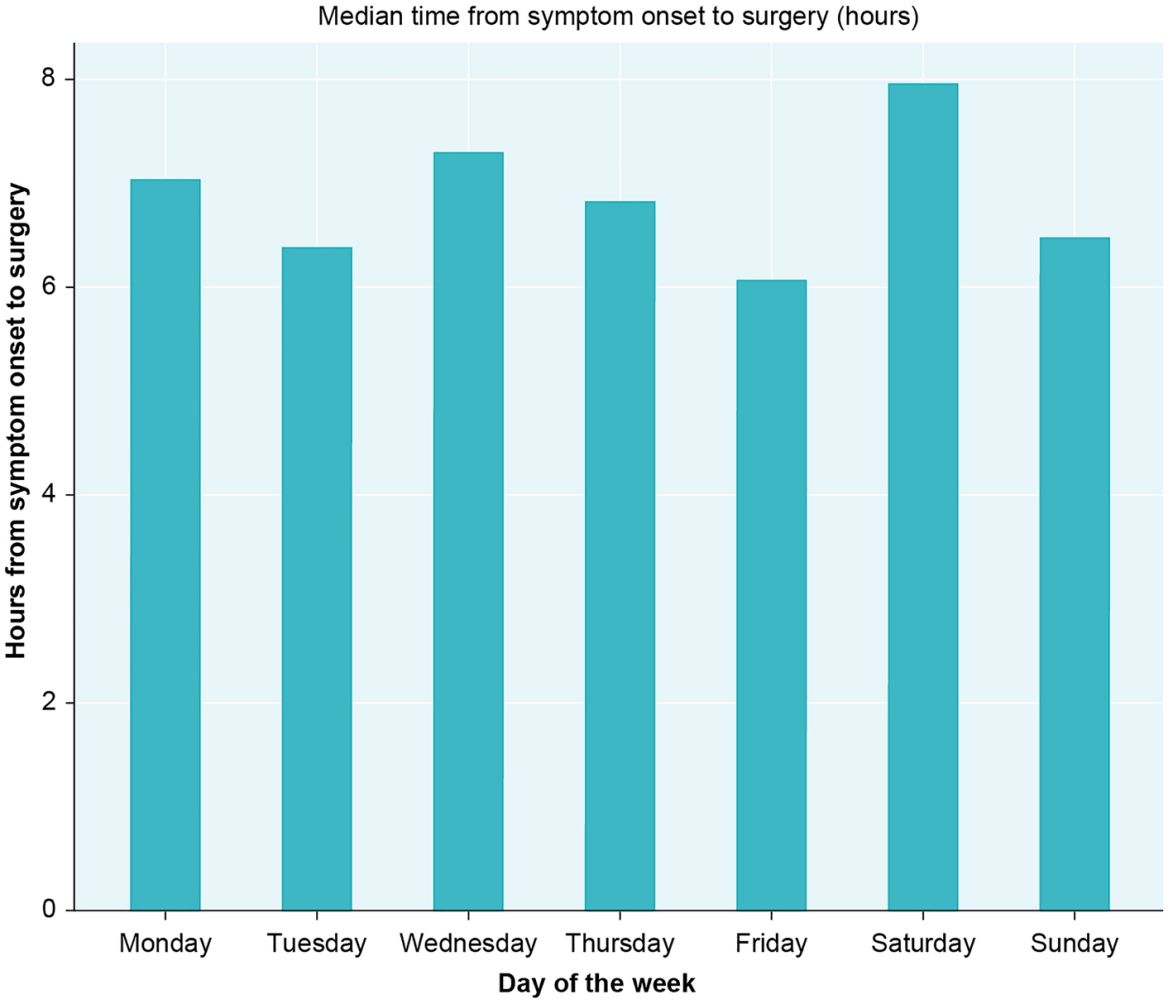
Median time between symptom onset and surgery on the different days of the week.

In addition, we performed post-hoc analyses to investigate whether ATAAD operations that should have been performed on weekends were carried forward to the following Monday, thus potentially explaining the observed lower incidence of ATAAD repair during weekends. Complete data on date and time of symptom onset as well as date and time of surgery were available for 799 of patients. We observed no differences in median waiting time between day of the week (ranging from 6.1 h on Fridays to 7.9 h on Saturdays, *p* = 0.40) (Fig. 2). Similar analyses were performed on national holidays and again, no statistically significant differences were observed (8.6 h vs 6.8 h, *p* = 0.13). There were no systematic differences in day of week distribution of operations between the group of patients with available data on symtom duration compared to remaining patients (*p* = 0.60). As there may be systematical differences between patients with and without data available on waiting time, we reran the analyses on the 799 patients only. The pooled results for weekends were similar (RR 0.74; 95% CI 0.64–0.92). Too few events were observed during national holidays to justify such an analysis.

For national holidays, the E-value and the E-value for the upper bound of the confidence interval were 2.2 and 1.3, respectively. For weekends, these E-values were 2.0 and 1.7, respectively.

## Discussion

In this study, we observed that national holidays and weekends were associated with a statistically significant reduction of operations for ATAAD compared to other working days.

Our findings regarding reduced incidence of ATAAD operations during national holidays and weekends contradict a previous study by Mehta et al. (2002), where no day of week patterns were observed in approximately 1000 cases of ATAAD^3^, but also a study by Zhang et al. (2018) from China where no effect was reported in a similarly sized patient cohort^5^. Adding to the discrepancy of results, Sumiyoshi et al. (2002) reported that Mondays, morning hours, and winter months had higher rates of acute aortic dissections, concluding that there were clear circadian and seasonal variations of AAD^4^. Most of these studies, however, were based on smaller and less homogenous samples than in the current study.

Among all known risk factors for ATAAD, hypertension is the most well-defined and important. It is present in almost 90% of patients and responsible for more than half of the population-attributable risk of aortic dissection^12^. Furthermore, aortic dissections are frequently triggered by increases in blood pressure^13^. Therefore, one may speculate that differences in patient blood pressure would explain our findings. However, in a study of more than 56,000 individuals (17,000,0000 measurements), only small differences in blood pressure between weekdays and weekends were observed (mean systolic blood pressure was 131.2 on weekdays versus 130.7 mm Hg on weekends)^14^, and thus, the variations described are most likely too small to be clinically relevant^14,15^. However, these studies did not take into account variations on blood pressure triggered by physical exertion, which may differ between working days, national holidays and weekends.

In other types of cardiovascular disease, Mohammad et al. (2018) reported a lower incidence of myocardial infarction on Saturdays and Sundays compared to other weekdays but also higher rates of myocardial infarction during Christmas and Midsummer holidays in Sweden^1^. Acute myocardial infarction is a complex and multifactorial condition, usually not triggered by temporary changes in blood pressure^16^, and therefore, other mechanisms may be responsible for the incidence patterns of ischaemic heart disease. Therefore, we speculate that the lower rates of ATAAD repair during national holidays and weekends may be related to other lifestyle confounders^16^.

Furthermore, we did not observe any differences between winter months and the rest of the year on the incidence of ATAAD during weekends and national holidays. Thus, we found nothing to support that short-term temperature extremes had an impact on the observed associations, in line with Chen et al. who reported no statistically significant association between temperature changes between consecutive days and the onset risk of acute aortic dissection^17^.

Since this study only includes patients operated for ATAAD rather for the event itself (up to 50% of the patients do not survive long enough to reach a hospital)^18^, it may be argued that the reduction in ATAAD surgeries during national holidays and weekends may be an effect of surgeons being less inclined to perform procedures during these days. However, ATAAD is a hyperacute condition and all participating centres adhere to the routine management of immediately performing surgery regardless of time of day or day of the week. This was supported by our analysis of cases where duration of symtoms was recorded, where no difference was observed between symptom onset and surgery on the day after a weekend or national holiday compared to other days. However, although we believe it to be highly unlikely, our analyses can not theoretically rule out that operations for ATAAD are more often turned down by surgeons on national holidays and weekends. Although a previous NORCAAD report did not demonstrate any differences in surgical outcomes between weekdays and weekends^19^, we cannot rule out that staffing patterns on different days of week and on national holidays may impact the level of experience of the attending surgeon. Furthermore, one could speculate that patients may be less inclined to seek medical attention while celebrating national holidays and weekends or that reduced healthcare resources during these days result in a larger proportion of ATAAD patients dying before being evaluated for surgical management. This, of course, would require further analyses based on large population registries. As discussed above, not only the fact that up to 50% of the patients succumb to the disease before reaching the hospital^18^ may have impacted our results. Another important factor may be misdiagnosis at the hospital preventing or delaying correct treatment. Recently Zaschke et al. (2020) reported that as many as up to 78% of patients may be initially misdiagnosed, but it has also been shown that the use of aortic dissection detection risk scores could improve the identification of patients at risk of aortic dissection and reduce the proportion of misdiagnosed patients^20^.

One of the main strengths of the current study is the long and well-defined time series of daily counts of aortic dissection data from the 10 university hospitals. Furthermore, we reported the time of surgery for ATAAD rather than the time of diagnosis. However, ATAAD is an acute condition and surgery is routinely performed without delay. Our data from the NORCAAD registry has shown that the median time between diagnosis and surgical treatment is seven hours. Finally, as with all retrospective studies, unmeasured confounders may have an impact on the results. However, we could show that an unmeasured confounder would have to be associatied with both the exposure and the outcome by a relative risk of approximately twofold each for both national holidays and weekends in order to discard our observation. We find the presence of such a strong confounder to be highly unlikely. Lastly, the study is isolated to centres from Northern Europe, and although the homogenicity of the study population strengthens this study, our results may not be generalisable to other regions. It should also be mentioned that only surgically treated patients were included in the study and, therefore, our study cohort does not account for the overall incidence of ATAAD. Furthermore, the hospitals' catchment areas vary significantly, which means that transport times can be anywhere from minutes to several hours, which in turn can affect the proportion of operated patients. On the other hand, all included centres share the principle that all patients who are not excluded for ATAAD repair due to co-morbidity and advanced age should be operated on urgently.

## Conclusion

In this study, the incidence of surgery for acute type A aortic dissection was significantly lower on national holidays and weekends compared to other days. Our analyses suggest that this observation is not explained by potential effects caused by seasonal differences or surgical delay only. However, the causality between national holidays, weekends and the decreased incidence of ATAAD requires further investigation, preferably including not only surgically treated patients.

## Data Availability

The datasets generated during and/or analysed during the current study are available from the corresponding author on reasonable request.
